# Rare Double Aneuploidy (Down-Klinefelter Syndrome): A Case Report

**DOI:** 10.7759/cureus.31330

**Published:** 2022-11-10

**Authors:** Mossa N Al Motawa, Majed J Al Buali, Amnah A Al Agnam, Abdulazeem A Alibraheem, Hussain T Bu Zaid

**Affiliations:** 1 Pediatric Endocrinology, King Faisal General Hospital, Al-Ahsa, SAU; 2 Pediatric Endocrinology, Diabeter Al-Ahsa, Al-Ahsa, SAU; 3 Pediatrics, King Faisal General Hospital, Al-Ahsa, SAU; 4 Family and Community Medicine, Primary Health Care Corporation, Al-Ahsa, SAU; 5 Pediatric Gastroenterology, King Faisal General Hospital, Al-Ahsa, SAU; 6 Neonatology, Maternity and Children's Hospital, Al-Ahsa, SAU

**Keywords:** klinefelter's syndrome, down's syndrome, 48xxy+21, diabetes mellitus, down-klinefelter syndrome, double aneuploidy

## Abstract

Double aneuploidies, such as Down syndrome and sex chromosome aneuploidies, are relatively rare. One rare form of double aneuploidy, Down-Klinefelter syndrome, is described here. The phenotypic characteristics of a three-year-old child showed the presence of features typical of Down syndrome. He had a global developmental delay, small testes, and diabetes mellitus by 18 months of age. Regardless of the presenting clinical features, karyotyping should be performed in all patients with suspected Down syndrome. In Down-Klinefelter syndrome, anticipatory phenotype goes beyond the sum of individual syndromic characteristics.

## Introduction

Chromosome aneuploidy refers to a cytogenetic disorder characterized by abnormalities in chromosome numbers. Aneuploidy is one of the most common and clinically significant chromosomal disorders. It occurs in about 0.3% of all liveborns, with the single most common abnormality being trisomy 21 [1].

It is possible to have a double aneuploidy on both sex and autosome chromosomes. There is no evidence that this is an inherited disorder. It can result from nondisjunction during meiosis I or meiosis II. In spite of this, the presence of two extra chromosomes in the same individual is rare [2].

Trisomy 21, also known as Down syndrome (DS), is a chromosomal disorder caused by the presence of a third copy of the 21st chromosome. Based on the updated national birth prevalence estimates for selected birth defects in the United States, DS was observed as having the second highest prevalence after clubfoot [3].

There are three main types of DS. They are free trisomy 21 (95%), translocation (5%), and mosaicism (2%). Other types, such as DS with a sex chromosome aneuploidy and DS with a translocation other than chromosome 21, are rare among the three main types [4].

Klinefelter syndrome (KS) is a group of chromosomal disorders that occur when there is an extra X chromosome in a typical male karyotype 46,XY. It is the most common sex chromosome disorder in humans, with a prevalence of one in 500 males [5]. While the incidence of KS is still unknown, the incidence of DS among the Saudi population is one in 554 [6,7].

DS is the most common genetic cause of intellectual disability, with most affected individuals having mild to moderate disabilities. Approximately 10% have autism spectrum disorder (ASD), and 6% have attention-deficit-hyperactivity disorder (ADHD). In addition to short stature, muscle hypotonia, and atlantoaxial instability (1-2%), individuals with DS often have reduced neuronal density, cerebellar hypoplasia, and congenital heart defects (50%) (in particular, atrioventricular septal defects). There is also an increased risk of developing certain health conditions, including hypothyroidism (1%), autoimmune diseases, diabetes mellitus (1.4-10.6%), sleep apnea (54-90%), epilepsy (8%), hearing and vision problems, hematological disorders (transient myeloproliferative disorder (TMD), also known as transient leukemia of DS, occurs in 5-30% of individuals with DS before three months of age), recurrent infections, anxiety disorders, and early-onset Alzheimer disease [4].

The characteristic features of KS syndrome are hypospadias, small phallus, and cryptorchidism in infancy. Developmental delays are common in toddler boys, especially with expressive language skills. Language delays, learning disabilities, and behavioral/social difficulties may be observed in school-aged children. In older children or adolescents, pubertal development may be delayed or incomplete. They may have a eunuchoid body habit, gynecomastia, and small testes. Adults may present with infertility or breast malignancy. In affected individuals, arms and legs are longer, and their height increases most significantly between the ages of five and eight [5].

The occurrence of double aneuploidy, the combination of two disorders in the same individual, is extremely rare. Ford et al. first described it in 1959. Most commonly, Down syndrome and Klinefelter syndrome occur together. In this case, the karyotype examination revealed 48 chromosomes. As a result, the patient had XXY sex constitution as well as an extra somatic chromosome characteristic of Down syndrome [8,9].

A combination of DS and other sex chromosome aneuploidies (other than XXY Klinefelter) has rarely been identified in patients with XXX, XXXY, XXY (Klinefelter), and monosomy X (Turner) [10].

Before 1959, Klinefelter syndrome was thought to be an endocrine disorder whose etiology was unknown, but Jacobs et al. recognized it to be a chromosomal disorder with an extra X chromosome [11].

A few cases of 48,XXY,+21 present with cardiac defects, but the majority present with typical Down syndrome features [12]. They also have a normal height. In one article, there was a child with hypothyroidism and undescended testes [13].

Presented here is a case report of a three-year-old boy with Klinefelter's syndrome and Down's syndrome due to possibly two meiotic nondisjunction events.

## Case presentation

The patient was born preterm at 27 weeks gestation with a birth weight of 1320 g (below the third percentile), and scored eight and nine on the Apgar score at one and five minutes, respectively. His parents, a 39-year-old father and a 40-year-old mother, were healthy unrelated parents as this was their second child. The first child is six years old and healthy. Due to low birth weight, the child stayed in the nursery for almost three months. A dysmorphological sign of Down syndrome led to the suspension of trisomy 21. The facial features include a flat face, up-slanting palpebral fissures, a flat nasal bridge with an epicanthal fold, and a relatively large tongue and low-set ears. He had mild generalized hypotonia but good suckling and swallowing.

In terms of weight, he continued to grow slightly above the 10th percentile, but above the 75th percentile for height using growth charts for children with DS. The most recent measurements were 11kg and 90cm at three years.

Neonatal screenings for congenital hypothyroidism, heart disease, and other diseases were negative. Abdominal and cranial ultrasound imaging were normal. Both the ophthalmological examination and the echocardiogram were unremarkable.

**Figure 1:**
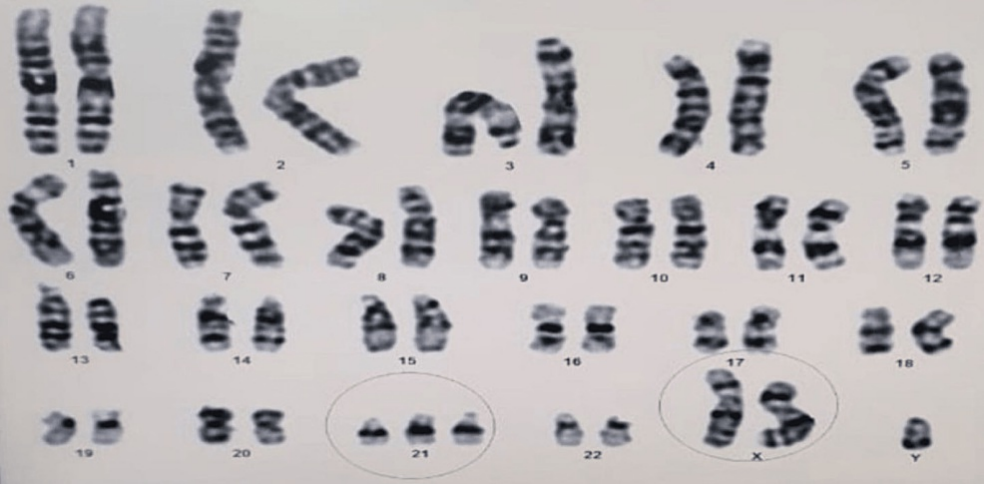
Complete karyotype of the child with Down-Klinefelter syndrome from peripheral blood lymphocytes revealed 48,XXY,+21

**Figure 2:**
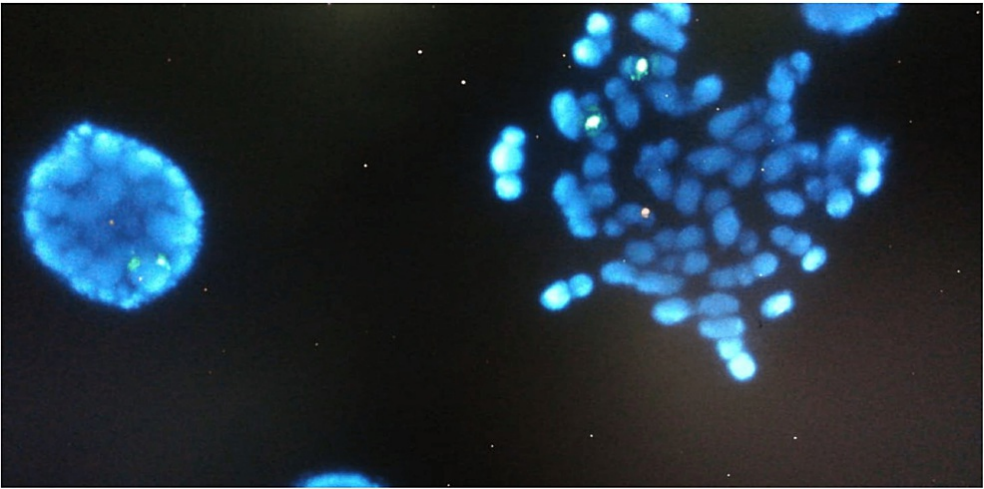
A FISH analysis showed three copies of sex chromosome (two copies of X chromosome and one copy of Y chromosome), and three copies of chromosome 21 FISH - fluorescence in situ hybridization

Analyses of the chromosomes and karyotypes of peripheral blood lymphocytes revealed 48, XXY,+21 (Figures 1 and 2). The parent chromosomal karyotyping has provided a normal result.

After discharge from the nursery, the proband was on a regular feeding and had scheduled follow-up appointments at the Down syndrome clinic. At 18 months, the patient developed diabetes mellitus when he presented with excessive sweating and polyuria for five days. He was admitted to the pediatric intensive care unit (PICU) for ten days due to diabetic ketoacidosis (DKA). He was managed then with multiple subcutaneous insulin injections in the form of insulin aspart two units pre each meal, and detemir six units in the morning and four units in the evening. The initial glycated hemoglobin was 11.5%. Further evaluation in the endocrine clinic revealed that his testes were descended but small in size. He had normal penile length. Testicular volume using Prader orchidometer was < 1ml on both sides (-1.5 SD; normal left: 1.56ml and right: 1.58 ml). Stretched penile length (SPL) was 3.5 cm (-1.5 SD). Testicular volume using ultrasonographic measurements showed right testis: 0.38ml, and left testis: 0.41ml (-1.0 SD; normal: 0.51ml). He had normal basal gonadotropins but a low basal testosterone level. He showed blunted response to the human chorionic gonadotrophin (HCG) stimulation test. Basal luteinizing hormone (LH) was 0.30 mIU/mL, follicle-stimulating hormone (FSH) was 0.20 mIU/mL, estradiol (E2) was 52 pg/ml, total testosterone was 0.087 nmol/L, and stimulated testosterone was 0.087 nmol/L (the normal response is an increase in testosterone level to ≥5.6nmol/l). The results suggest impaired testicular function, but further assessment at puberty is warranted [14].

**Figure 3:**
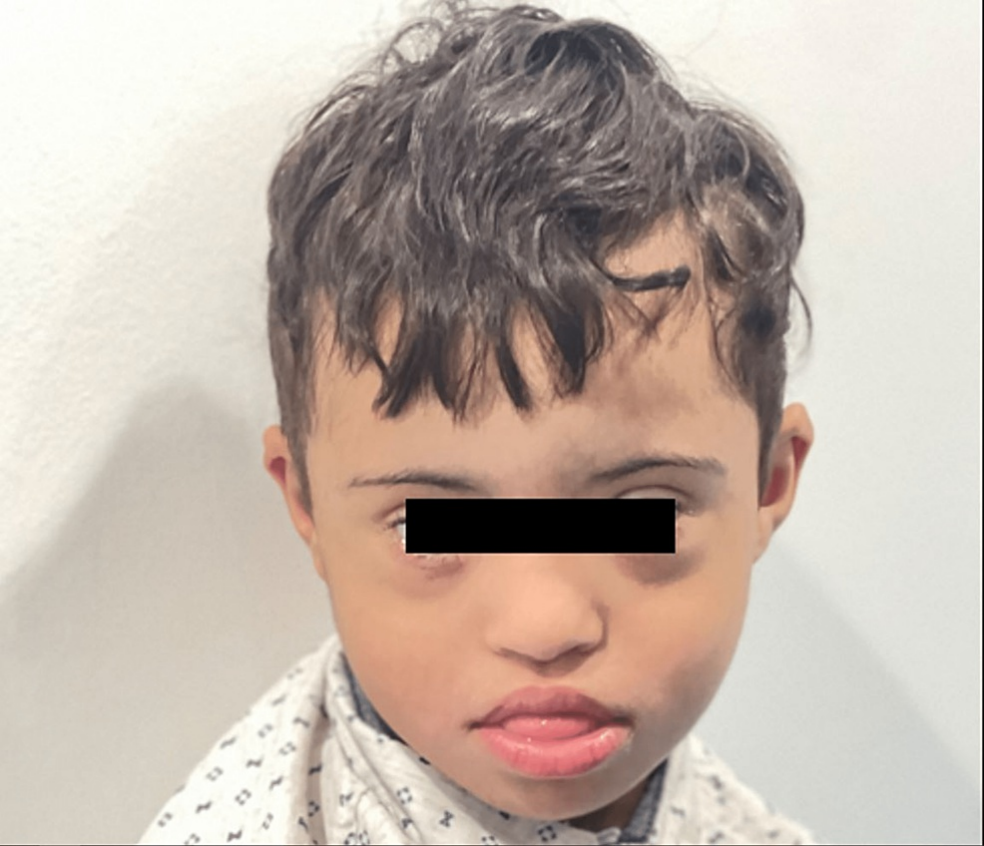
Frontal photograph of a three-year-old boy with Down-Klinefelter syndrome showing typical facial features of Down syndrome; flattened nose, upward slanting eyes, small ears, small mouth, and protruded tongue

**Figure 4:**
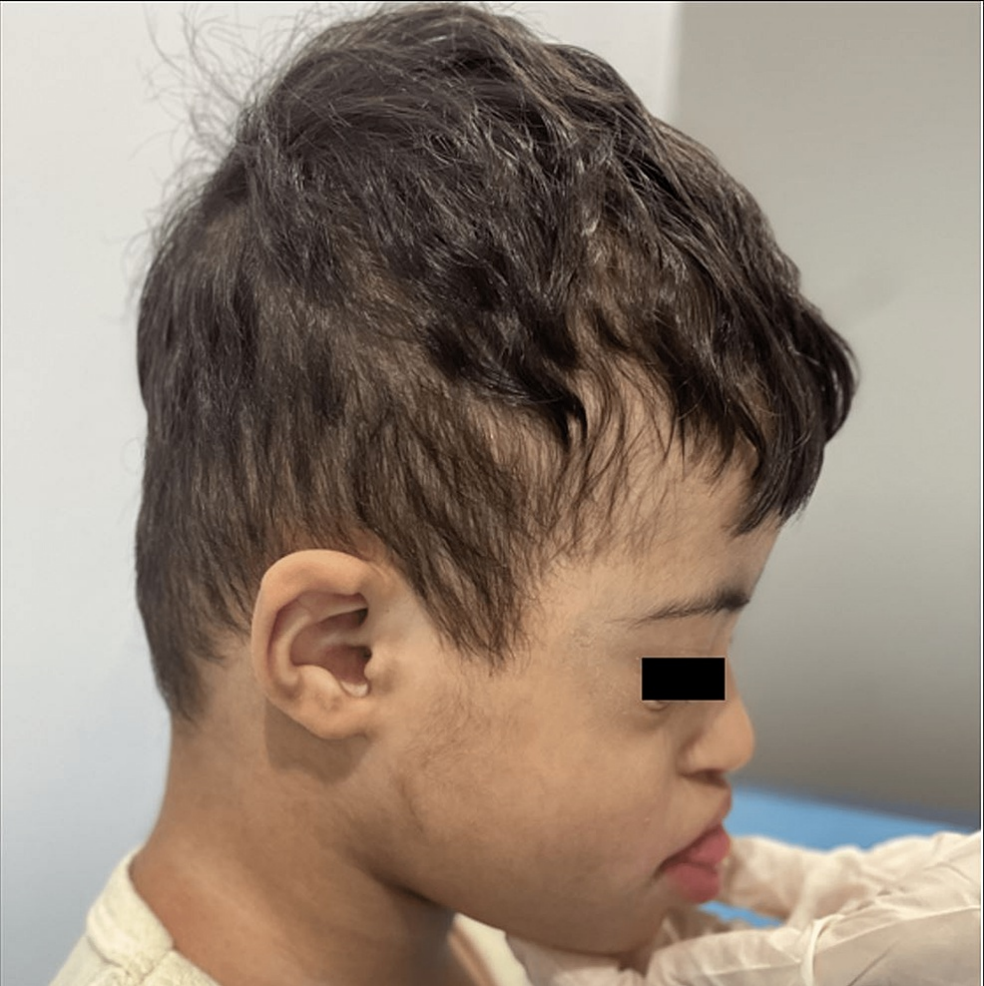
Lateral photograph of the patient with Down-Klinefelter syndrome showing low set and small ear, flat occiput, and protruded tongue

**Figure 5:**
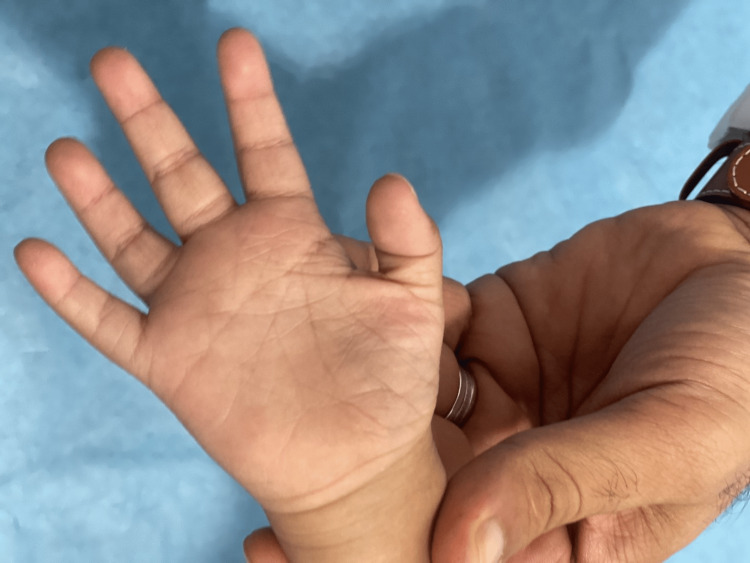
Photograph of the left hand of the patient with Down-Klinefelter syndrome showing transverse palmar crease (simian crease): a single line that runs across the palm of the hand

Developmental delays affecting all aspect of milestones was evident in the subsequent visits. As per the last outpatient visit (30 months old), he can only sit without support, lifts his chest on extended arms, rolls front to back, creep but can’t crawl, and was unable to stand when pulled. He can transfer objects, palmar grasp, shake rattles, and mouth objects but can't scribble or look for fallen toys. He turns to sound and says vowels and syllables but can't say 'mama' or 'dada'. He understands 'no' and 'bye bye' but can't point to a named body part or drink from a cup (Figures 3, 4, 5).

Cytogenetic analysis

Karyotyping

Chromosomal karyotyping was performed on lymphocytes derived from peripheral blood. A total of 20 cells were counted at the metaphases using Giemsa banding by trypsin, bands per haplotype set (BPHS) 450. Analyzing and karyotyping six cells of metaphases was performed.

Fluorescence In Situ Hybridization (FISH)

We used the fluorescence in situ hybridization (FISH) probe set: locus-specific identifier (LSI) SO DNA probe hybridizes to 21q22.13-q22.2 for chromosome 21 chromatin studies and LSI CEP X/SRY for sex determination according to standard procedures.

## Discussion

As the name implies, aneuploidy is the abnormal number of chromosomes. This condition is typically observed in older mothers, though double aneuploidy occurs rarely. When there is a double aneuploidy, there is usually one sex chromosome and one autosomal chromosome aneuploidy. The condition usually manifests as a double trisomy [10].

Early in the course of double aneuploidy, the clinical picture is almost always compatible with autosomal chromosome aneuploidy. It is evident from this case and most published reports of 48,XXY,+21 that they exhibit features typical of Down syndrome alone, since the features of Klinefelter syndrome are not evident until after pubertal development, as one would expect [15].

Individuals with hypogonadism can be classified based on where the primary defects are located in their hypothalamic-pituitary-gonadal (HPG) axis. A primary defect at the level of the gonads is termed hypergonadotropic hypogonadism (primary gonadal failure), whereas hypogonadotropic hypogonadism results from abnormalities of the hypothalamus and pituitary gland [16]. Turner syndrome and Klinefelter syndrome are the two most common causes of hypergonadotropic hypogonadism.

KS adversely affects testicular function. Boys with KS may have micropenis or cryptorchism at birth as a result of intrauterine hypogonadism. But, the majority of boys with Klinefelter syndrome appear similar to boys with normal karyotypes, so the disorder is typically discovered as adults when infertility or gynecomastia is a common symptom [15,17].

During the first three months, it is possible to detect hypergonadotrophic hypogonadism if the mini-puberty is blunted. Cryptorchidism and micropenis, however, were present in our case, so such disorders were not evaluated. At age two, he showed blunted response to HCG stimulation. In these cases, thorough evaluation at puberty is necessary, since they may have elevated levels of gonadotrophins; follicle-stimulating hormone (FSH), luteinizing hormone (LH), and estradiol (E2) with low levels of testosterone, and the testes may grow a little but shrink subsequently [15].

Androgen replacement therapy may help normalize body proportions or facilitate the development of normal secondary sex characteristics at puberty. Testosterone also improves behavior and work performance, but its effects are limited in treating infertility, gynecomastia, and small testes [5].

Patients with Down syndrome are ten times more likely to suffer from type 1 diabetes than those without, with prevalence rates between 1.4 and 10.4 percent. Compared to most individuals with Down syndrome reported in the literature, the proband developed diabetes mellitus (DM) at an earlier age. Unlike our case, patients with Down syndrome are generally older than ten years when they develop type 1 DM, according to Anwar et al. [18].

The risk of thyroid disease increases with age, as does the risk of autoimmune-related thyroid dysfunction [4]. However, there is no evidence of thyroid disease in our case, but one should monitor these children periodically.

The Down-Klinefelter syndrome presented in our case was characterized by the clinical features of Down syndrome and the early onset of diabetes mellitus. As far as we know, nearly 70 cases of 48,XXY,+21 trisomies have been reported worldwide, none of which developed diabetes.

## Conclusions

Double trisomy with 48,XXY,+21 chromosomes is a rare chromosomal abnormality. The patient exhibits characteristic features of Down syndrome at birth, while features of Klinefelter syndrome might not be apparent until puberty. In all suspected Down syndrome patients, confirmatory cytogenetic testing is recommended. Early childhood diabetes may develop in patients with Down-Klinefelter syndrome.
